# Autonomic and Cognitive Function Response to Normobaric Hyperoxia Exposure in Healthy Subjects. Preliminary Study

**DOI:** 10.3390/medicina56040172

**Published:** 2020-04-10

**Authors:** Sławomir Kujawski, Joanna Słomko, Karl J. Morten, Modra Murovska, Katarzyna Buszko, Julia L. Newton, Paweł Zalewski

**Affiliations:** 1 Department of Hygiene, Epidemiology, Ergonomics and Postgraduate Training, Division of Ergonomics and Exercise Physiology, Collegium Medicum in Bydgoszcz, Nicolaus Copernicus University in Torun, 85-094 Bydgoszcz, Poland; jslomko@cm.umk.pl (J.S.); p.zalewski@cm.umk.pl (P.Z.); 2 Nuffield Department of Women’s and Reproductive Health, University of Oxford, Oxford OX3 9DU, UK; karl.morten@wrh.ox.ac.uk; 3 Institute of Microbiology and Virology, Riga Stradiņš University, LV-1067 Riga, Latvia; modra.murovska@rsu.lv; 4 Department of Theoretical Foundations of Bio-Medical Science and Medical Informatics, Collegium Medicum, Nicolaus Copernicus University, 85-067 Bydgoszcz, Poland; buszko@cm.umk.pl; 5 Institute of Cellular Medicine, The Medical School, Newcastle University, Framlington Place, Newcastle-upon-Tyne NE2 4HH, UK; julia.newton@newcastle.ac.uk

**Keywords:** oxygen therapy, physical medicine, oxidative stress, cognitive function

## Abstract

*Background and objective*: This is the first study to investigate the effect of high-flow oxygen therapy, using a normobaric chamber on cognitive, biochemical (oxidative stress parameters and the level of neurotrophins), cardiovascular and autonomic functioning. *Materials and methods*: 17 healthy volunteers, eight males and nine females, with a mean age of 37.5 years, were examined. The experimental study involved ten two-hour exposures in a normobaric chamber with a total pressure of 1500 hPa (32–40 kPa partial pressure of oxygen, 0.7–2 kPa of carbon dioxide and 0.4–0.5 kPa of hydrogen). Cognitive function was assessed by using Trail Making Test parts A, B and difference in results of these tests (TMT A, TMT B and TMT B-A); California Verbal Learning Test (CVLT); Digit symbol substitution test (DSST); and Digit Span (DS). Fatigue (Fatigue Severity Scale (FSS)), cardiovascular, autonomic and baroreceptor functioning (Task Force Monitor) and biochemical parameters were measured before and after intervention. *Results*: After 10 sessions in the normobaric chamber, significant decreases in weight, caused mainly by body fat % decrease (24.86 vs. 23.93%, *p* = 0.04 were observed. TMT part A and B results improved (*p* = 0.0007 and *p* = 0.001, respectively). In contrast, there was no statistically significant influence on TMT B-A. Moreover, decrease in the number of symbols left after a one-minute test in DSST was noted (*p* = 0.0001). The mean number of words correctly recalled in the CVLT Long Delay Free Recall test improved (*p* = 0.002), and a reduction in fatigue was observed (*p* = 0.001). Biochemical tests showed a reduction in levels of malondialdehyde (*p* < 0.001), with increased levels of Cu Zn superoxide dismutase (*p* < 0.001), Neurotrophin 4 (*p* = 0.0001) and brain-derived neurotrophic factor (*p* = 0.001). A significant increase in nitric oxide synthase 2 (*p* = 0.02) and Club cell secretory protein (*p* = 0.015) was also noted. Baroreceptor function was significantly improved after normobaric exposures (*p* = 0.003). Significant effect of normobaric exposures and BDNF in CVLT Long Delay Free Recall was noted. *Conclusions*: This study demonstrates that 10 exposures in a normobaric chamber have a positive impact on visual information and set-shifting processing speed and increase auditory-verbal short-term memory, neurotrophic levels and baroreceptor function. A response of the respiratory tract to oxidative stress was also noted. There is a need to rigorously examine the safety of normobaric therapy. Further studies should be carried out with physician examination, both pre and post treatment.

## 1. Introduction

Oxygen is essential for life and is involved in generating energy via mitochondrial respiration. Increasing the partial pressure of O_2_ in inspired air is potentially an effective therapeutic option in disease (i.e., neurological conditions) and increasing performance in sport. Oxygen therapy has been used for almost a century to treat a range of medical conditions including emergency medicine to patients in intensive care units (ICU). Studies have primarily focused on hyperbaric hyperoxia, even though its widespread use has been difficult due to limited access to hyperbaric chambers. In experimental studies, normobaric hyperoxia when used to treat acute ischemic stroke patients can prevent tissue death from ischemia, reduce cerebral ischemic injury and improve functional outcome [1]. Studies show that normobaric hyperoxia improves cerebral blood flow and oxygenation [2]. Side effects of the treatment are few with minor disorders associated with pulmonary surfactant secretion in some patients [3].

Recently oxygen supplementation has been shown to improve performance in elite athletes [4]. Three mechanisms have been proposed to account for the benefits of hyperoxia in sport: (1) direct application of hyperoxia during exercise increasing tissue oxygenation; (2) hyperoxia used after exercise to improve brain function and allowing faster recovery; and (3) hyperoxia may increase and maintain training effects. 

The optimal concentration of oxygen to provide the greatest benefit in a particular experimental setting is currently unclear. Inhaled oxygen concentrations ranging from 30% to 100% have been studied, with no direct comparison of the impact of different oxygen concentrations on the physiological response [4]. Generally, hyperbaric oxygen therapy (HBOT) requires application of pressure of three atmosphere absolute (3 ATA). Inhaling oxygen at 3 ATA increases the partial pressure of oxygen in the blood to >200 kPa, increasing the oxygen concentration in arterial blood from 6.6 to 6.8 mL (O_2_/100 mL) [5]. However, the side effects of Hyperbaric Oxygen Treatment (HBOT) in rare cases include oxygen toxicity, pulmonary edema and toxicity, and more commonly hyperoxic myopia [6]. Moreover, many patients experience claustrophobia in the chamber and it has been suggested that reduced chamber be used in order to reduce side effects [6].

To our knowledge, this is the first study to assess hyperoxia treatment of healthy subjects with partial pressure 32–40 kPa of oxygen under normobaric conditions (1500 hPa) in an environment less likely to cause claustrophobia. The aim of this study was to explore the effectiveness of a program consisting of 10 sessions of high-flow oxygen therapy on cognitive, biochemical (oxidative stress parameters and the level of neurotrophins), cardiovascular and autonomic functioning in a normobaric chamber. 

## 2. Materials and Methods

### 2.1. Setting

This study took place between September 2018 and December 2018 and was approved by the Ethics Committee, Ludwik Rydygier Memorial Collegium Medicum in Bydgoszcz, Nicolaus Copernicus University, Torun (KB 700/2018); written informed consent was obtained from all of the participants. 

### 2.2. Study Group

Twenty-three participants were initially enrolled in the study. Three declined to take part, and a further three subjects were excluded; two experienced unpleasant/painful ear congestion while undergoing exposure, while the final subject had an underlying medical condition which had not been known before. Results from seventeen healthy volunteers, 8 males and 9 females, with a mean age of 37.5 years, were available for analysis. All potential study participants were questioned about their health state, sleep quality and life habits. All participants had a high level of education, had successfully completed a recent physician examination and did not suffer from any known underlying medical conditions. Some patients were taking the contraceptive pill. An initial Cardiopulmonary Exercise Test (CPET) and spirometry showed no evidence of an abnormality. 

All subjects in the period preceding the study (3 days before) and during normobaric treatment were advised not to change diet or levels of physical activity. It was also recommended that they maintained a similar pattern of sleep and activity hours, avoiding extreme physical efforts and emotionally burdensome situations. The main exclusion criteria for subjects included the following: shift work, participation in sports at competitive level, alcohol consumption within 12 h before the test, receiving any medication and diet supplements during the study and potential disorders of the cardiovascular or respiratory system observed during initial testing.

### 2.3. Intervention—Ten Normobaric Exposures

**Figure 1:**
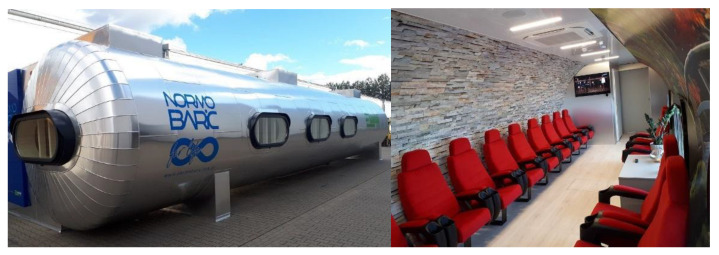
Normobaric chamber (Type V120K1, Ekonstal).

The experimental study was performed in a normobaric chamber (V120K1 type, Ekonstal, Poland; Figure 1), which consists of two compartments: the antechamber and the proper chamber, which were connected by a door. Ekonstal is the only producer of a fully equipped and CE certified normobaric chamber in Poland. A pressure of 1500 hPa was maintained in the normobaric chamber, with 32–40 kPa partial pressure of oxygen, 0.7–2 kPa of carbon dioxide and 0.4–0.5 kPa of hydrogen. Participants underwent a total 10 two-hour normobaric chamber exposures over a period of 10 days (from Monday to Friday, scheduled at the same time each day, one session per day).

### 2.4. Body Composition Analysis

To measure body composition analysis, a multi-frequency bioelectrical impedance analyzer (Tanita MC-180MA Body Composition Analyzer, Tanita UK Ltd., Middlesex, UK) was used. Patients’ skin was cleaned with a sanitizer and sterile dressing from each limb’s parts that are in contact to each of the 4 electrodes. Participants were instructed to hold grips with electrodes on the level of their hips with arms straight and kept sideways and slightly away from the trunk. All of the measurements were at the same time of day, under consistent conditions.

### 2.5. Cognitive Function and Fatigue Measurements

The California Verbal Learning Test (CVLT) measures episodic verbal learning and memory [7]. The examiner reads aloud list A, consisting of 16 nouns in a fixed order, with a one-second interval between each word. The same list was read five times. After each list reading, the subject is asked to recall as many words as they can remember. After five repetitions of list A, participants are exposed to an interference list (list B) and asked to recall it. Then, the rest of the cognitive tests were conducted, which took 20 min. After 20 min, participants were asked to recall list A again. Results of free and cued recall of list A were tested immediately (Short Delay Free Recall), and again, after 20 min (Long Delay Free Recall), they were analyzed.

The Trail Making Test (TMT) provides information on visual search, scanning, speed of processing, mental flexibility and executive functions. The TMT consists of two parts. Trail Making Test part A (TMT A) is a test of visual processing speed that requires an individual to draw lines sequentially, connecting 25 encircled numbers distributed on a sheet of paper. Trail Making Test part B (TMT B) is similar, except the person must alternate between numbers and letters (e.g., 1, A, 2, B, 3, C, etc.). Overall, the result of TMT B is an indicator of visual processing speed and executive function/set-shifting performance. The score on each part links to the time required to complete the task [8]. The difference between the scores in part B and A is an indicator of executive function/set-shifting performance and is denoted as TMT B-A.

The Digit symbol substitution test (DSST) involves the transcription of a digit-symbol code, using a key over a set time period. The amount of correct decoding is indicative of processing speed [9]. The output of the test is given as the number of symbols remaining un-coded after a 60 s test.

The dDigit Span Test (DST) involves listening to a sequence of numbers and to repeat them. In Digit Span Forward (DSF), participants are asked to repeat the sequence in in ascending order, while in Digit Span Backward (DSB), they repeat it in reverse order. If two sequences of the same length are correctly repeated, then the examiner moves to next, longer set of digits. Overall, results of the DSF and DSB, and the sum of these two results, are taken into statistical analysis [9]. DSB requires the storage of data for a short period of time, while the string of digits is rotated. DSB requires efficient functioning of the working memory, while the DSF result is closely related to effectiveness of auditory attention [9].

Fatigue symptoms were measured by using the Fatigue Severity Scale [10].

### 2.6. Cardiovascular Measurements

Hemodynamic (heart rate (HR), systolic blood pressure (sBP), diastolic blood pressure (dBP), mean blood pressure (mBP), stroke index (SI), cardiac index (CI) and total peripheral resistance index (TPRI)) and autonomic parameters (low frequency (LF), high frequency (HF), LF/HF and total slope mean) were automatically measured at rest and during an active standing test (AS), with a Task Force Monitor®, TFM (CNS Systems, Gratz, Austria). The Task Force Monitor® is designed for noninvasive measurements of hemodynamic parameters and consist of electrocardiography (ECG), impedance cardiography (ICG), and oscillometric (oscBP) and continuous (contBP) blood pressure measurement [11].

Total slope mean, linked to heart function, was calculated by the Task Force Monitor software, using a sequence method which relies on the selecting sequences of four or more consecutive heart cycles co-occurring with either a progressive increase in systolic blood pressure and R–R interval or by a progressive decrease in systolic blood pressure and R–R interval. The total slope mean of the regression line between systolic blood pressure and R–R interval changes (both increase and decrease) was calculated and served as an index of the sensitivity of arterial baroreflex modulation of heart rate [12,13]. 

### 2.7. Biochemical Parameters

Blood collected in the antecubital vein was used for biochemical ELISA analyses (HBSS, Immuniq, Zory, Poland): Nitric oxide synthase 2 (NOS-2), Nitric oxide synthase 3 (NOS-3), Malondialdehyde (MDA), Neurotrophin-4 (NT 4), Neurotrophin-3 (NT 3), brain-derived neurotrophic factor (BDNF), copper zinc superoxide dismutase—CuZn-SOD (SOD1) and club cell secretory protein (CC16) 

Measurements of the above parameters were obtained at 2 points during the study, at baseline, before 10 exposures and after 10 exposures in normobaric chamber (after sessions).

### 2.8. Cardiopulmonary Exercise Testing and Spirometry

The cardiopulmonary exercise test (CPET) test was performed in the presence of a physician, using the Bruce protocol (Cardiovit CS-200 Ergo-Spiro, Schiller AG, Baar, Switzerland) [14]. Before each test, a brief instruction of walking on treadmill was provided. A trained technician advised every participant that the test would end when anaerobic threshold was reached. Each test was carried out in the same air-conditioned room, with constant temperature between 20 and 22 °C, and relative humidity at 50–60% Anaerobic threshold (AT) was calculated on the assumption that the respiratory exchange ratio (RER) = 1. 

Spirometry using the same equipment was provided before each CPET.

### 2.9. Statistical Analysis

A Shapiro–Wilk test was used to assess normality of the data. Variables where values did not show a normal distribution were analyzed by using Wilcoxon signed-rank test, to compare data before and after intervention; otherwise, a *t*-Test was used. Mean value and standard deviation (SD) are reported, and a significance level was set on 0.05. The statistical package STATISTICA 13.1 (StatSoft, Inc.) was used to carry out the data analysis. Mixed models with random effects based on a two-way ANOVA were used to test the effect of an intervention (time effect denoted as before (before intervention) and after (after intervention)) on cognitive function and biochemical parameters. Mixed models with random effects (based on the maximum likelihood method used to estimate variance parameters) were applied in order to determine the time dependence of parameters. Analyses were performed with R version 3.5.0 (R: library lme) and Matlab 2017b [15]. Violin graphs were created with a ggstatsplot library, to show the dynamic of changes in values of single patients in response to normobaric therapy [16]. 

## 3. Results

Table 1 shows the mean value of anthropometric parameters and body composition. After 10 sessions in the normobaric chamber, significant decreases in weight and body fat percentage were observed. 

**Figure 2:**
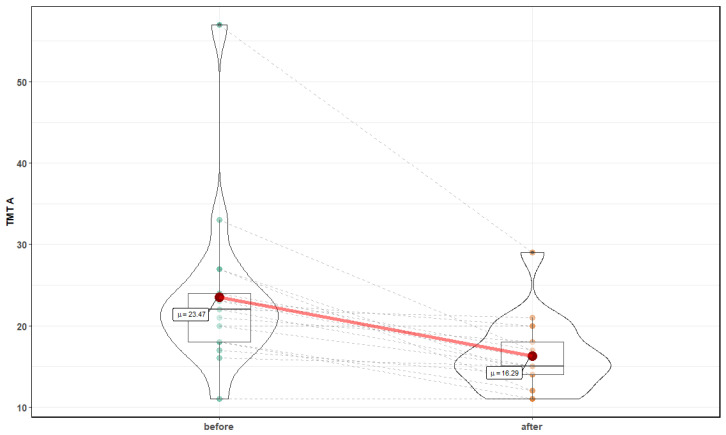
Effects of normobaric therapy on Trial Making Test part A. Red dots connected by red line indicates mean value; horizontal black line inside the box denotes median value. Green dots before, and orange dots after, connected by dashed lines, denote results of individual patients. Shape of violin graph indicates distribution of results.

**Figure 3:**
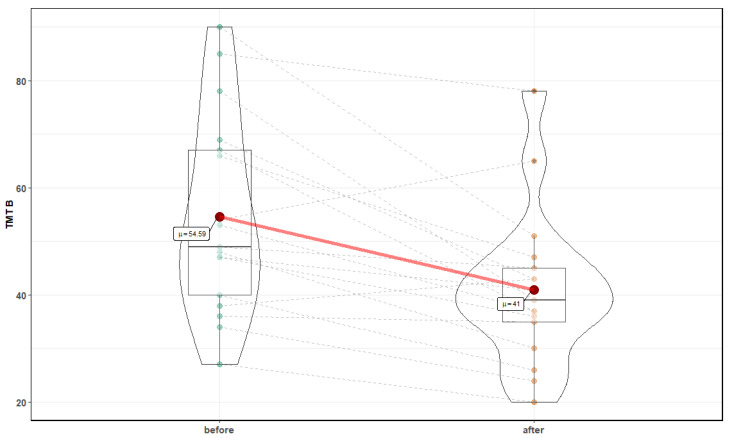
Effects of normobaric therapy on Trial Making Test part B.

Both parts of the TMT assessment showed a significant improvement following normobaric exposures. Figure 2 and Figure 3 show changes after 10 sessions in normobaric chamber in part A (23.5 ± 10 s before vs. 16.3 ± 4.5 after, Z = 3.41, *p* = 0.0007) and part B (54.59 ± 18.4 s before vs. 41 ± 14.3 after, T = 4, *p* = 0.001, respectively). There were no statistically significant changes in TMT B-A, suggesting lack of improvement in executive function.

**Figure 4:**
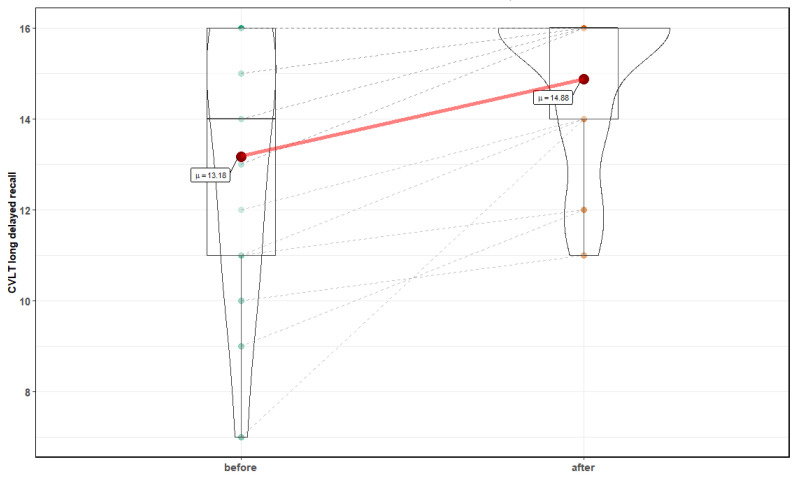
Effects of normobaric therapy on CVLT long delayed recall.

Normobaric exposures improved performance in the DSST assessment test. There was a significant decrease in the average number of symbols left after the 60 s test, with an average of 53.9 ± 8.7 symbols left before normobaric exposures, compared to 48.1 ± 7.2 after treatment, T = 5, *p* = 0.0001). Changes in DSST 12.53 ± 12.8 symbols left after 120 s before vs. (6.88 ± 9.6 after intervention, were nonsignificant (*p* = 0.22). In the CVLT Long Delay Free Recall test, there was a significant improvement in mean number of words correctly recalled after normobaric exposures, with 13.18 ± 2.8 words recalled before, compared to 14.88 ± 1.7 after, Z = 3.06, *p* = 0.002) (Figure 4). Changes in CVLT Short Delay Free Recall were nonsignificant, with an additional 1.5 word recalled after normobaric exposures, Z = 1.95, *p* = 0.054. However, no significant improvement in any of three analyzed results of DS test were noted. 

**Figure 5:**
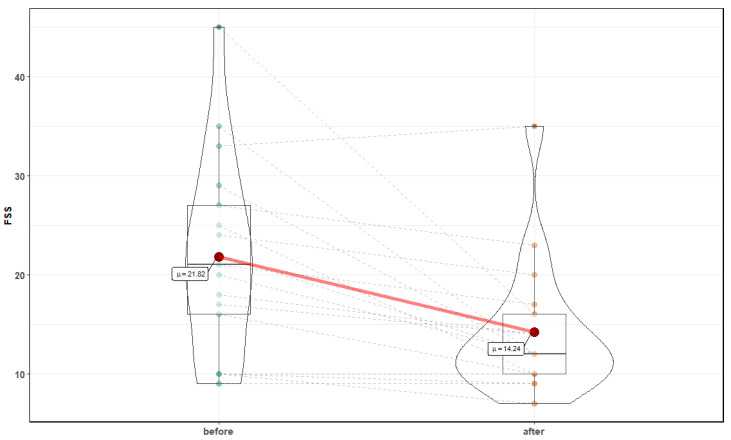
Fatigue Severity Scale before and after 10 normobaric exposures.

Moreover, a significant reduction in FSS was noted (21.82 ± 10 before vs. 14.24 ± 6.8 after, Z = 3.29, *p* = 0.001) (Figure 5).

**Figure 6:**
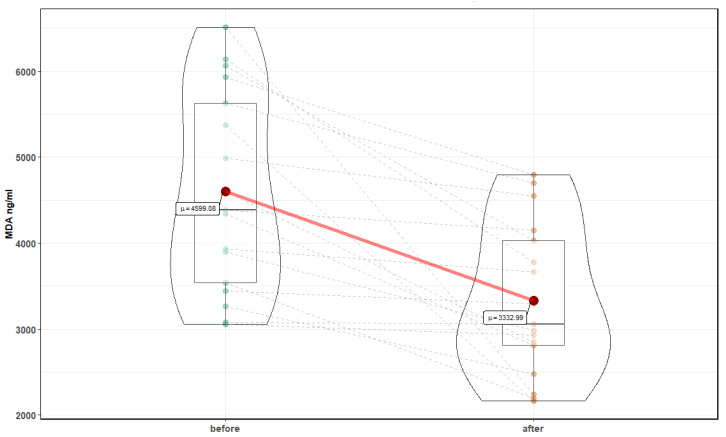
MDA before and after 10 normobaric exposures.

**Figure 7:**
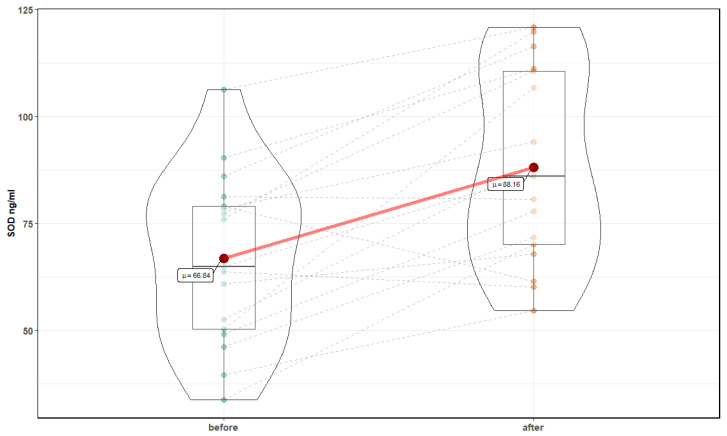
SOD before and after 10 exposures in normobaric chamber.

**Figure 8:**
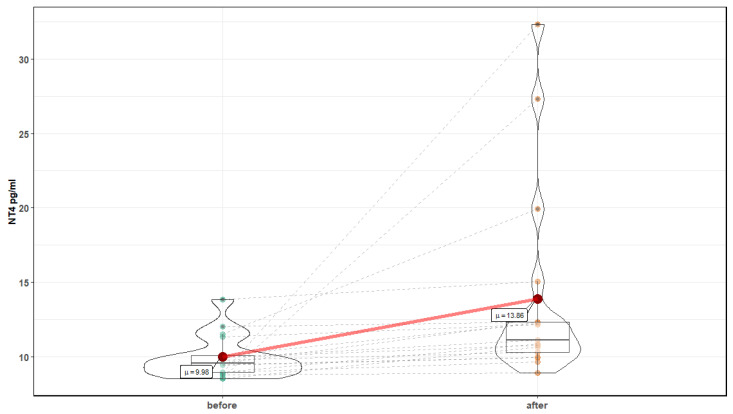
NT 4 before and after 10 exposures in normobaric chamber.

**Figure 9:**
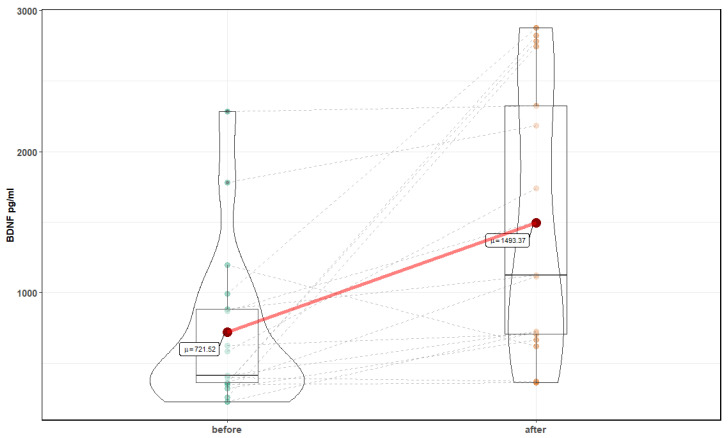
Effects of normobaric therapy on BDNF.

After 10 sessions in the normobaric chamber, significant changes in markers of oxidative stress were noted: a reduction in the level of MDA, 4599.1 ± 1166.4 ng/mL before, compared to 3332.99 ± (880.4 after (T = 4.43, *p* <0.001), and an increase in the level of Cu Zn SOD 66.84 ± 19.8 ng/mL before compared to 88.16 ± 22.4 after (T = 4.78, *p* < 0.001) (Figure 6 and Figure 7). In addition, a significant increase in the level of neurotrophins was noted: NT 4 and BDNF were observed. NT4 showed an increase for 9.98 ± 1.4 pg/mL before, compared to 13.86 ± 6.6 after (Z = 3.82, *p* = 0.0001), and BDNF 721.52 ± 574.8 before pg/mL to 1493.37 ± 943.1 after (Z = 3.26, *p* = 0.001) (Figure 8 and Figure 9). Changes in NT 3 and NOS-3 were not statistically significant. There was a statistically significant increase in the level of NOS-2, 214.52 ± 48 pg/mL before, compared to 272.6 ± 131.2 after (Z = 2.29, *p* = 0.02). CC16 significantly increased 5.25 ± 2.2 ng/mL before compared to 6.89 ± 2.2 after (T = 2.71, *p* = 0.015).

CVLT Short Delay Free Recall results measures of episodic verbal learning and memory are presented in Table 2. In linear mixed model with random effects fir by maximum likelihood normobaric exposures had no effect on CVLT Short Delayed Free Recall (*p* = 0.34), but BDNF significantly affected this test result (*p* = 0.0062). 

In Table 3, results of the linear mixed model with random effects for CVLT Long Delay Free Recall as dependent variable are presented. The results indicate that there was a significant effect of normobaric exposures (*p* = 0.017) and BDNF (*p* = 0.021). 

We also performed the linear mixed models for improved cognitive function tests, to assess the effects of normobaric exposures, MDA, LF/HF or BDNF as independent variables. Here, normobaric exposures significantly affected the cognitive test (*p* < 0.05), while the other variables mentioned above showed no significant effects 

There were no statistical differences in hemodynamic parameters. Total slope mean (baroreceptor function) after normobaric exposures was significantly higher, at 17.91 ± 9.1 before, compared to 22.13 ± 9.2 after treatment (T = 3.47, *p* = 0.003) (Table 4). No significant changes in response to the active standing test were noted (Table 5). 

No significant changes in CPET and spirometry results were noted (*p* > 0.05).

## 4. Discussion

The major finding of the this study is that 10 exposures in a normobaric chamber with total pressure of 1500 hPa, in combination with 32–40 kPa partial pressure of oxygen, 0.7–2 kPa of carbon dioxide and 0.4–0.5 kPa of hydrogen, have a positive impact on the processing speed of visual information and set-shifting, auditory-verbal short-term memory, increases neurotrophin expression and enhances baroreceptor functioning. A reduction in the levels of MDA and an increase in the level of SOD might be considered to be positive influences on the ability of the individual to deal with oxidative stress. However, an increase in levels of CC16 might be considered to be an indicator of increased oxidative stress and inflammation in respiratory system. Under some circumstances, raised SOD2 levels are indicative of oxidative stress, although the levels might be related to signaling [17]. No significant changes in cardiopulmonary exercise and spirometry results were noted. 

Neurotrophins (NT4 and BDNF) play a crucial role in neuroplasticity, neurogenesis and neuroprotection in the central nervous system [18]. Both NT4 and BDNF showed an increase in plasma levels after normobaric exposures, and this may link with the improved CVLT results, which showed a significant improvement in both short and delayed free recall. However, in the case of CVLT, no alternate version was used, as there was no available alternate version in Polish; therefore, small practice effect might have occurred, explaining the observed results [19]. 

Consistent with our results, serum BDNF levels also correlated with good scores on the short form of the Boston Naming Test in healthy older adults [20]. An aerobic physical exercise program also resulted in a raised plasma BDNF level [21]. Further studies to understand how the biological changes caused by normobaric therapy link to the improvement in cognitive function improvement would be particularly valuable. A significant improvement was noted in both of the visual processing speed tests (TMT A and DSST). Performance on the DSST could be influenced by associative learning, potentially explaining the observed similarities in the pattern of cognitive improvement [22]. In contrast, no significant changes in Digit Span test were noted. However, previous studies have reported high correlation levels (r = 0.55) between the DSB result and fluent intelligence [23]. It cannot be assumed that normobaric sessions will affect all aspects of cognitive functioning. Fluid intelligence does not appear to be influenced by normobaric exposures

Baroreceptor function was affected by normobaric exposure, with changes also observed in the regulation of sympatho-parasympathetic balance and blood pressure regulation in response to an active standing test, suggesting improvement in the control of orthostatic reactions with potential heart health benefits. Therefore, normobaric therapy could be considered as adjunctive therapy for orthostatic intolerance. Further clinical studies would include a study of changes in signals complexity analysis due to normobaric therapy [24,25]. 

Normobaric chamber exposures also resulted in modulation of oxidative stress, as evidenced by significant changes in biochemical parameters: MDA and SOD. The results might suggest improved local tissue flow and a reduction in the intensity of oxidative stress linked to reduced levels of free radicals. However, increased level of CC16, an indicator of respiratory system oxidative stress and inflammation [26], was also noted after normobaric therapy. Hyperoxia has been previously shown to worsen arterial function due to raised free radical levels and the induction of transient endothelial dysfunction [27]. Importantly, the increase in SOD observed in the above study might be the response to increased oxidative stress linked to oxygen-induced ROS. CC16 levels were reduced in patients with lung injury in comparison to healthy participants [28]. Kurowski et al. demonstrated that a high-intensity physical-exercise program is related to a decrease in serum CC16 levels, which in turn makes the respiratory tract more prone to infections and associated impaired lung function [29]. In sarcoidosis, CC16 was proposed to be a marker of the integrity of the air–blood barrier [30]. Other studies considering the link between normobaric hyperoxia and oxidative stress are controversial, with the choice of experimental model, disease state and duration of hyperoxia all influencing the results. In cell and tissue studies from the brain, lungs and kidney, increased oxidative stress is strongly associated with normobaric hyperoxia [31,32,33]. Other studies have investigated the effect of hyperoxia and oxidative stress in acute ischemic stroke, traumatic brain injury and multiple sclerosis. Here, studies show that brief or continual normobaric hyperoxia exposures for seven days did provide evidence of oxidative damage. In these diseases, some authors suggest that the benefits of the hyperoxia sessions in improving cell and tissue function may outweigh the risk of potentially enhanced increased reactive oxygen species generation [34,35,36]. 

The observed changes in baroreceptor parameters in this study may suggest improvement of the mechanisms regulating orthostatic reactions and cardioprotective activity even in an environment of increased oxidative stress. This observation is in line with restoration of decreased baroreflex sensitivity after short-term exposure to oxygen in type 1 diabetes patients [37]. Our other interesting finding that normobaric sessions appear to modulate body weight by reducing body fat mass is intriguing and needs to be followed up in a larger study

Acute responses to hyperoxic breathing on occurrence in the euphoric state in some participants and improvement of absolute power output in a subsequent exhaustive exercise test were noted [38]. Other authors show that breathing fraction of inspired oxygen 31.35% induces changes in perceived exertion during physical exercise, by delaying the decrease in cerebral oxygenation during maximal exercise [39]. Our results show that hyperoxia exposures with 32–40 kPa partial pressure of oxygen under normobaric condition (1500 hPa) does not affect the functioning of the cardiovascular system. The observation of changes in sympatho-vagal balance during the active standing test after normobaric chamber exposure supports previous studies suggesting that 100% oxygen at 2.5 ATA improves parasympathetic system [40]. Autonomic changes are potentially mediated by a reduced input from peripheral chemoreceptors to the medulla oblongata. Moreover, significant changes in baroreceptor parameters and a trend toward changes of oxide synthase (NOS-2) after sessions in the normobaric chamber may serve as evidence of improvement in the mechanisms regulating orthostasis and cardioprotective effects. Under physiological conditions, nitric oxide provides a constant vasodilator tone against sympathetic vasoconstriction. In hyperoxia conditions, NO synthesis can be stimulated to provide a mechanism for the regulation of vascular tone. Other authors suggest that forearm vasoconstriction is observed during hyperbaric hyperoxia but not under normoxia at 1 ATA [41]. In line with our results, other studies have noted that hyperoxic breathing reduces fatigue, boosts subjective perception of energy level and increases alertness [42]. Normobaric hyperoxia has proved to be effective in modulating microcirculation in healthy subjects [43]. Some results suggest that functional changes in the operation of the brain result from changes in cerebral blood flow induced by hyperoxia [44,45]. In research exploring the effect of hyperoxia on the fMRI signal, alternation of resting state network activity has been observed [46].

A practical implication from this study is the need for a clinical examination before normobaric therapy, to monitor patients during treatment with follow up after therapy. As some patients resigned from our study due to side effects, there is a need for clinical monitoring of future studies of normobaric exposures in order to ensure safety.

One limitation of this study is the relatively small number of participants. Most of the available studies of hyperoxia and its influence on autonomic nervous system have been conducted on small groups and investigating only the influence of one of two variables (i.e. O_2_ or pressure). Future studies in the normobaric chamber should investigate a range of gas variables (e.g., variations in levels of, oxygen, carbon dioxide and hydrogen) and its impact upon various disease states. The risk to benefit ratio of therapy in the normobaric chamber should also be evaluated. In addition, a control group undergoing the same intervention without hyperoxia was not included in this study; we suggest the blinding of participants in future studies, in order to exclude any placebo effects. However, proper sham-intervention in control group based on exposure to 1.2 ATA would elevate plasma oxygen by circa 30%, as compared to normal pressure [47]. It could be hypothesized that even a small amount increase of ATA could result in brain function alteration [48]. Results of randomized, controlled clinical trials have provided evidence of significant improvement of nervous system function due to the room air at 1.3/1.2 ATA [48,49]. Moreover, 1.3 ATA seems to be the smallest pressure change sensed by humans [50]. Therefore, a lower ATA would be not adequate stimuli in the sham intervention group, because patients could be aware of belonging to the sham group. 

As patients were not followed up post-normobaric-exposure, information as to whether benefits were sustained long-term are not available. Furthermore, lack of alternative cognitive testing after the intervention did not allow us to control for the possible influence of the learned/practice effect on the results. Therefore, we would propose that our preliminary study should be extended to increase the size of group and add an appropriate sham-intervention group with follow-up examination included. In addition future studies should be carried out assessing the impact of normobaric therapy on clinical groups where fatigue, cognitive impairment and dysautonomia are significant problems.

## 5. Conclusions

The major finding of the present study is that 10 exposures in a normobaric chamber have a positive impact on processing speed of visual information and set-shifting, baroreceptors functioning and oxidative stress. Increased neurotrophins expression and improvement in some aspects of visual processing speed, set-shifting, long delayed free recall of auditory verbal stimuli and reduction of fatigue were observed. Moreover, an increase in the level of neurotrophins (NT4 and BDNF) was observed. Normobaric chamber exposures also resulted in a reduction of oxidative stress, as evidenced by significant changes in biochemical parameters, MDA and SOD. We observed an increase in SOD and a decrease in MDA level, which could suggest improved local tissue flow and reducing the intensity of oxidative stress and thus probably lowering the free radical reaction and strengthening the antioxidant defense. Importantly, the increase in SOD observed in the above study might be the response to increased oxidative stress potentially caused by oxygen-induced ROS or enhanced signaling. Moreover, an increased level of CC16 may reflect adaptation of pulmonary endothelium to oxidative stress. Other studies considering the association between normobaric hyperoxia and oxidative stress generate conflicting results as a consequence of inconsistencies in experimental models, differences in disease state and the duration of hyperoxia exposures. 

In addition, changes of baroreceptor parameters may suggest improvement of the mechanisms regulating orthostatic reactions and cardioprotective activity. 

In summary, exposures in a normobaric chamber seem to be able to improve some domains of cognitive function, neurotrophic factors, oxidative stress and autonomic regulation in healthy subjects. However, physician examination before normobaric therapy and progress monitoring are strongly advised.

## Figures and Tables

**Table 1:** Values of anthropometric parameters and body composition before and after 10 exposures in normobaric chamber. BMR = basal metabolic rate; FatP = body fat percentage; FFM = Fat-free mass; VFatL = Visceral Fat Level.

Parameter	Before Mean ± SD	After Mean ± SD	*p*-Value
Weight (kg)	81.44±23.5	81.01±23.3	0.03
BMR (kcal)	7498.97±1944.2	7543.56±1970.4	0.35
FatP (%)	24.86±7.8	23.93±7.7	0.04
FFM (kg)	60.32±15.5	60.77±15.6	0.31
VFatL	6.88±5.3	6.65±4.9	0.18
Bone Mass (kg)	3.00±0.7	3.04±0.7	0.08

**Table 2:** Results of linear mixed model with random effects for CVLT Short Delay Free Recall. The time (normobaric exposures) and BDNF are the independent variables. SE = standard error.

	Value	SE	*p*-Value
Intercept	11.81	0.64	<0.01
before:after	0.61	0.63	0.34
BDNF	0.0015	0.0005	0.006

**Table 3:** Results of linear mixed model effects fit by maximum likelihood for CVLT Long Delay Free Recall. The time and BDNF are the independent variables. SE = standard error.

	Value	SE	*p*-Value
Intercept	12.45	0.56	<0.0001
before: after	1.22	0.46	0.017
BDNF	0.0009	0.0004	0.021

**Table 4:** Mean values of cardiovascular and autonomic parameters (at rest) before and after 10 exposures in normobaric chamber. HR = heart rate; sBP = systolic blood pressure; dBP = diastolic blood pressure; mBP = mean blood pressure; SI = stroke index; CI = cardiac index; TPRI = total peripheral resistance index; HF = high-band frequency spectrum; LF = low-band frequency spectrum.

Parameter	Before Mean ± SD	After Mean ± SD	*p*-Value
HR (1/min)	69.77 ± 11.2	68.46 ± 10	>0.05
sBP (mmHg)	114.26 ± 16.4	113.38 ± 14.4	>0.05
dBP (mmHg)	74.25 ± 9.0	74.61 ± 8.5	>0.05
mBP (mmHg)	90.58 ± 10.8	90.50 ± 9.3	>0.05
SI (ml/m^2^)	51.71 ± 12	50.39 ± 13.3	>0.05
CI (l/min/m^2^)	3.51 ± 0.6	3.36 ± 0.7	>0.05
TPRI (dyn*s*m^2^/cm^5^)	2110.44 ± 664.4	2200 ± 582.1	>0.05
LF-RRI (ms^2^)	506.62 ± 475.1	708.18 ± 734.3	>0.05
HF-RRI (ms^2^)	410.35 ± 339.1	581.4 ± 580.6	>0.05
LF/HF	1.64 ± 1.2	1.76 ± 1.3	>0.05
LF-dBP (mmHg^2^)	5.54 ± 5.1	6.29 ± 5.8	>0.05
HF-dBP (mmHg^2^)	0.94 ± 0.9	1.41 ± 1.1	>0.05
LF-sBP (mmHg^2^)	8.25 ± 7.7	6.45 ± 4.7	>0.05
HF-sBP (mmHg^2^)	2.91 ± 3.6	2.49 ± 1.6	>0.05
Total Slope Mean (ms/mmHg)	17.91 ± 9.1	22.13 ± 9.2	0.003

**Table 5:** Magnitude of changes (delta) induced by active standing test on hemodynamic parameters before and after 10 exposures in normobaric chamber.

Parameter	Before Mean ± SD	After Mean ± SD	*p*-Value
Δ HR (1/min)	−14.50 ± 5.7	−13.64 ± 5.3	>0.05
Δ sBP (mmHg)	−17.63 ± 8.9	−16.51 ± 7.5	>0.05
Δ dBP (mmHg)	−19.95 ± 5.7	−18.96 ± 7.9	>0.05
Δ mBP (mmHg)	−18.82 ± 6.7	−17.46 ± 7.6	>0.05
