# Correction: Kujawski et al. Autonomic and Cognitive Function Response to Normobaric Hyperoxia Exposure in Healthy Subjects. Preliminary Study. *Medicina* 2020, *56*, 172

**DOI:** 10.3390/medicina60020255

**Published:** 2024-02-01

**Authors:** Sławomir Kujawski, Joanna Słomko, Karl J. Morten, Modra Murovska, Katarzyna Buszko, Julia L. Newton, Paweł Zalewski

**Affiliations:** 1 Department of Hygiene, Epidemiology, Ergonomics and Postgraduate Training, Division of Ergonomics and Exercise Physiology, Collegium Medicum in Bydgoszcz, Nicolaus Copernicus University in Torun, 85-094 Bydgoszcz, Poland; jslomko@cm.umk.pl (J.S.); p.zalewski@cm.umk.pl (P.Z.); 2 Nuffield Department of Women’s and Reproductive Health, University of Oxford, Oxford OX3 9DU, UK; karl.morten@wrh.ox.ac.uk; 3 Institute of Microbiology and Virology, Riga Stradiņš University, LV-1067 Riga, Latvia; modra.murovska@rsu.lv; 4 Department of Theoretical Foundations of Bio-Medical Science and Medical Informatics, Collegium Medicum, Nicolaus Copernicus University, 85-067 Bydgoszcz, Poland; buszko@cm.umk.pl; 5 Institute of Cellular Medicine, The Medical School, Newcastle University, Framlington Place, Newcastle-upon-Tyne NE2 4HH, UK; julia.newton@newcastle.ac.uk

## Text Correction

There was an error in the original publication [1]. Specific information regarding the gas pressures in the chamber was incorrectly reported.

A correction has been made to Abstract, 1. Introduction, 2. Materials and Methods (2.3. Intervention—Ten Normobaric Exposures), 4. Discussion (first and seventh paragraph). In addition, minor errors and repetitions have been corrected.

The experimental study involved exposure during ten two-hour periods in a normobaric chamber with a total pressure of 1500 hPa (32–40 kPa partial pressure of oxygen, 0.7–2 kPa of carbon dioxide, and 0.4–0.5 kPa of hydrogen).

The authors state that the scientific conclusions are unaffected. This correction was approved by the Academic Editor. The original publication has also been updated.
